# No Evidence for Neural Overlap between Unconsciously Processed and Imagined Stimuli

**DOI:** 10.1523/ENEURO.0228-21.2021

**Published:** 2021-10-12

**Authors:** Nadine Dijkstra, Simon van Gaal, Linda Geerligs, Sander E. Bosch, Marcel A. J. van Gerven

**Affiliations:** 1 Donders Institute for Brain, Cognition and Behaviour, Radboud University, 6500 GL, Nijmegen, The Netherlands; 2 Wellcome Centre for Human Neuroimaging, University College London, London WC1N 3AR, United Kingdom; 3 Department of Psychology, Brain & Cognition, University of Amsterdam, 1000 GG, Amsterdam, The Netherlands

**Keywords:** consciousness, mental imagery, perception, unconscious processing

## Abstract

Visual representations can be generated via feedforward or feedback processes. The extent to which these processes result in overlapping representations remains unclear. Previous work has shown that imagined stimuli elicit similar representations as perceived stimuli throughout the visual cortex. However, while representations during imagery are indeed only caused by feedback processing, neural processing during perception is an interplay of both feedforward and feedback processing. This means that any representational overlap could be because of overlap in feedback processes. In the current study, we aimed to investigate this issue by characterizing the overlap between feedforward- and feedback-initiated category representations during imagined stimuli, conscious perception, and unconscious processing using fMRI in humans of either sex. While all three conditions elicited stimulus representations in left lateral occipital cortex (LOC), significant similarities were observed only between imagery and conscious perception in this area. Furthermore, connectivity analyses revealed stronger connectivity between frontal areas and left LOC during conscious perception and in imagery compared with unconscious processing. Together, these findings can be explained by the idea that long-range feedback modifies visual representations, thereby reducing representational overlap between purely feedforward- and feedback-initiated stimulus representations measured by fMRI. Neural representations influenced by feedback, either stimulus driven (perception) or purely internally driven (imagery), are, however, relatively similar.

## Significance Statement

Previous research has shown substantial neural overlap between imagery and perception, suggesting overlap between bottom-up and top-down processes. However, because conscious perception also involves top-down processing, this overlap could instead reflect similarity in feedback processes. In this study, we showed that the overlap between perception and imagery disappears when stimuli are rendered unconscious via backward masking, suggesting reduced overlap between purely bottom-up and top-down generated representations.

## Introduction

Visual experience relies on neural representations in visual cortex, which can be activated in two different ways: externally, by light bouncing off objects and hitting the retina, from which signals are sent via feedforward connections to early visual cortex (EVC) and areas further up in the visual hierarchy [e.g., lateral occipital cortex (LOC)]; or internally, via feedback signals from high-level brain areas, such as areas in prefrontal cortex, for example, during mental imagery and dreaming (Mechelli et al., 2004; Dentico et al., 2014; Dijkstra et al., 2017b). It remains unclear to what extent activation patterns in visual cortex caused by feedforward and feedback signals are similar.

Previous work has compared neural representations during perception and imagery, revealing convincing evidence that there is neural representational overlap between perception and imagery throughout large parts of visual cortex (O’Craven and Kanwisher, 2000; Thirion et al., 2006; Stokes et al., 2009; Reddy et al., 2010; Cichy et al., 2012; Lee et al., 2012; Albers et al., 2013; Johnson and Johnson, 2014; Dijkstra et al., 2017a; Horikawa and Kamitani, 2017). The strongest overlap between perception and imagery is typically observed in high-level visual areas (Stokes et al., 2009; Reddy et al., 2010; Lee et al., 2012), whereas the overlap in low-level areas seems to depend on the required detail of the imagery task (Kosslyn and Thompson, 2003) and the experienced imagery vividness (Lee et al., 2012; Albers et al., 2013; Dijkstra et al., 2017a,b).

However, while activation in visual cortex during mental imagery indeed only relies on feedback signals (Mechelli et al., 2004; Dijkstra et al., 2017a,b, 2020), visual activation during perception reflects an interplay between feedforward and feedback processes (Lamme and Roelfsema, 2000; Muckli, 2010; Bastos et al., 2012, 2015; Dijkstra et al., 2017a,b, 2020). To determine whether visual representations activated by feedforward and feedback signals do indeed activate similar neural populations, one needs to investigate a situation in which visual representations are caused by feedforward signals only and compare those to events that include feedback processing as well.

Backward masking has been hypothesized to disrupt feedback from high-level visual cortex to early visual cortex (Lamme et al., 2002; Roelfsema et al., 2002; Del Cul et al., 2007; Fahrenfort et al., 2007; van Gaal and Lamme, 2012). In a backward-masking paradigm, a briefly presented target stimulus is rapidly followed by a second masking stimulus. Appropriate backward masking renders the target stimulus invisible. Several studies have shown that masking leaves the feedforward sweep relatively unaffected, which can still cause activation in high-level visual cortex (Jiang and He, 2006; Sterzer et al., 2008), while feedback processing is disrupted (Lamme et al., 2002; Fahrenfort et al., 2007; van Gaal and Lamme, 2012; Mashour et al., 2020). These and other observations have led to the idea that the feedforward sweep is unconscious and that recurrent processing is an important factor in achieving conscious awareness (Tononi, 2008; Lamme, 2015; Mashour et al., 2020). However, the exact relationship between feedback processing and conscious awareness is still debated (Boly et al., 2017).

**Figure 1.**
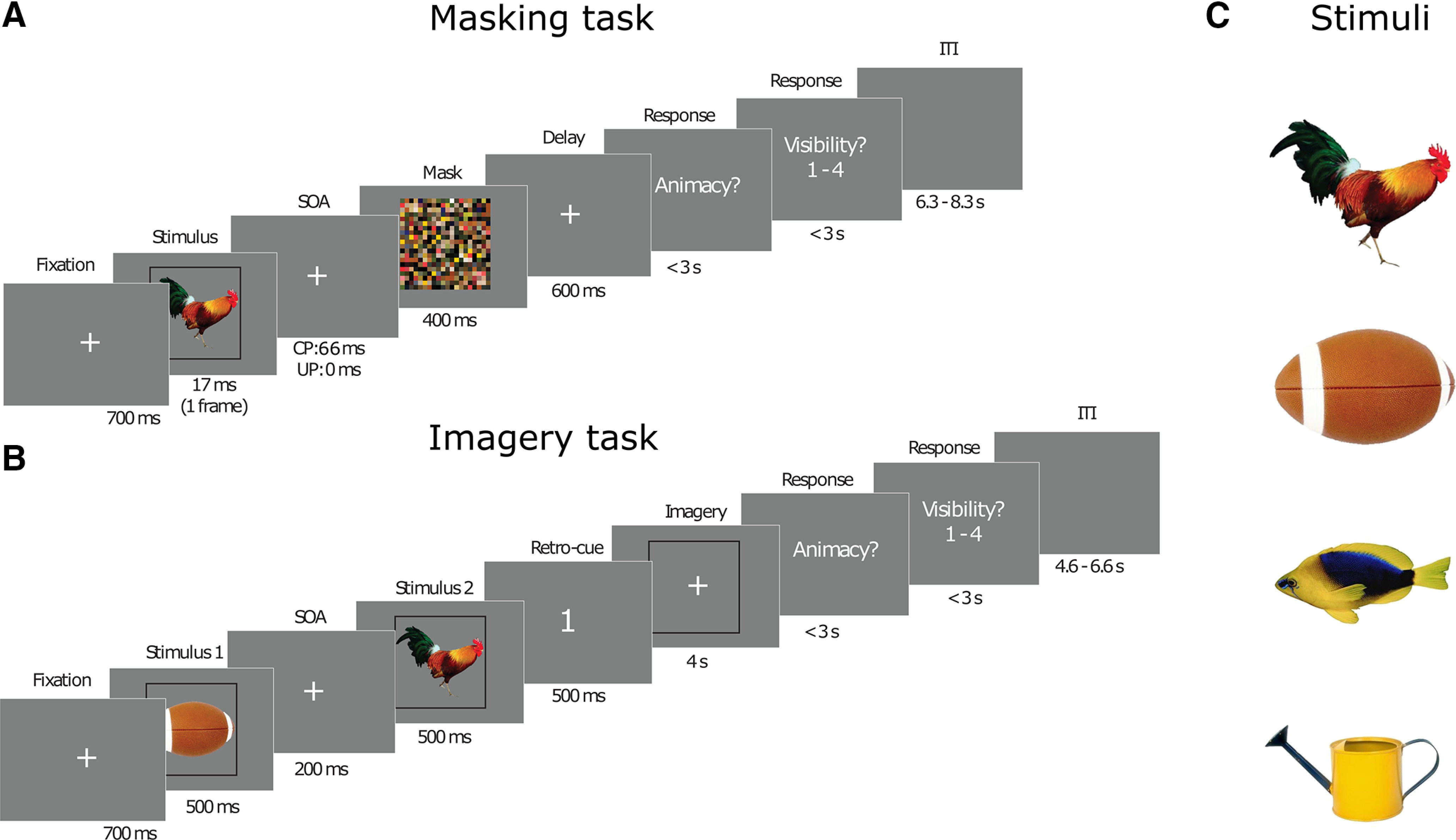
Experimental paradigm. ***A***, Masking task. A stimulus is presented for 17 ms, followed by a mask (duration, 400 ms) after 0 ms (unconscious condition) or 66 ms (conscious condition). Participants had to indicate whether the stimulus was animate or inanimate and rate the visibility. ***B***, Visual imagery task. Participants were presented with two stimuli after each other followed by a cue indicating whether to imagine the first or the second stimulus, as vividly as possible. After the imagery, participants had to indicate whether the imagined stimulus was animate or inanimate and rate the visibility of their imagery. ***C***, Stimuli used: a rooster, a football, a fish, and a watering can from the POPORO stimulus dataset (Kovalenko et al., 2012). The neural analyses focused on pairwise comparisons between all possible combinations of stimuli.

In the current study, we investigated to what extent visual representations in visual cortex are modified by feedback by comparing conditions in which stimuli are consciously perceived, not consciously perceived, and imagined. We rely on the assumption that unconscious processing contains less or no feedback processing, and that therefore comparing unconscious to conscious and imagined representations will provide insight into the effects of feedback processing. However, it is important to note that this is an assumption based on previous research that will not be tested in the current study. Therefore, the exact implications of our results need to be inferred with caution. More elaborate and nuanced interpretations will be given in the Discussion. We quantified the representational overlap between the different conditions by training a classifier on one condition and testing it on another condition (“cross-decoding”; Lee et al., 2012; Albers et al., 2013; Dijkstra et al., 2018). The only difference between the conscious and unconscious condition was the stimulus onset asynchrony (SOA) between the target and the mask. To cue visual imagery in a way that does not induce an informative cue signal that can be picked up by a classifier, we used a retro-cue paradigm (Harrison and Tong, 2009; Fig. 1*B*).

## Materials and Methods

### Participants

Thirty-seven participants with normal or corrected-to-normal vision gave written informed consent and participated in the study. All participants were naive to the aim of the experiment, and most participants were familiar with similar visual perception fMRI studies. Two participants were excluded from the final analyses: one because they quit the experiment prematurely and one because they had misunderstood the task. Because of an accidental change in the refresh rate of the monitor (from 60 to 75 Hz), the timing was slightly different for 6 of 35 participants [presentation from 17 to 13 ms, conscious interstimulus interval (ISI) from 66 to 80 ms], so that the presentation times were slightly shorter for the unconscious condition and slightly longer for the conscious condition. Because this error did not change visibility ratings [unconscious: 1.37 (SD = 0.27) vs 1.35 (SD = 0.58; *t*_(33)_ = 0.079, *p *=* *0.94); conscious: 2.92 (SD = 0.37) vs 2.98 (SD = 0.61; *t*_(33)_ = −0.25, *p *=* *0.80)] or discrimination sensitivity (unconscious: 0.19 (SD = 0.28) vs 0.03 (SD = 0.18; *t*_(33)_ = 1.9, *p *=* *0.07); conscious: 3.33 (SD = 0.61) vs 3.82 (SD = 0.90; *t*_(33)_ = −1.26, *p *=* *0.22)], we decided not to remove these participants. Thirty-five participants were included in the main analyses (mean age, 25.9 years; SD* *=* *5.9).

### Experimental design

Before the experiment, participants filled out the Vividness of Visual Imagery Questionnaire 2 (Marks, 1973), which is a 16-item questionnaire that measures the general vividness of a participant’s imagery. The experiment consisted of two tasks, a perception and an imagery task, which were executed in interleaved blocks, and whether participants started with the imagery or perception task was counterbalanced over participants. The perception task consisted of conscious and unconscious trials, which only differed in ISI between the stimulus and the mask: 0 ms for the unconscious trials and 66 ms (four frames) for the conscious condition. We chose to operationalize conscious versus unconscious processing via experimental manipulation (strong vs weak masking) and not via *post hoc* trial selection based on visibility reports, because this latter approach has been shown to violate statistical assumptions and may lead to spurious unconscious effects (for more details, see Shanks, 2017). During the perception task, a stimulus was presented very briefly (17 ms), followed by a backward mask. Participants subsequently indicated whether the presented stimulus was animate or inanimate and rated the visibility of the stimulus on a scale from 1 (not visible at all) to 4 (perfectly clear; Fig. 1*A*). To prevent motor preparation, the response mapping for both the animacy and visibility ratings were randomized over trials. During the imagery task, two stimuli were each successively presented for 500 ms, followed by a retro-cue indicating which of the two the participant should imagine. The participant then imagined the cued stimulus and subsequently indicated the animacy and the visibility of the imagined stimulus (Fig. 1*B*). The button-response mapping for the animacy task and the visibility rating was randomized over trials to prevent motor preparation.

There were 184 conscious and 184 unconscious trials, and 46 repetitions per stimulus, divided over four blocks. Each conscious–unconscious block lasted ∼9 min. There were 144 imagery trials, and 36 repetitions per stimulus, divided over four blocks. Each imagery block lasted ∼7 min. The order of the different stimuli and SOAs (unconscious vs conscious trials) within the perception task and the stimuli and retro-cue combinations during imagery was fully counterbalanced within participants, and which task (imagery or perception) was executed first was randomized between participants. In total, there were eight blocks, leading to an experimental time of ∼65 min/participant. Including breaks and an anatomic scan, this added up to 90 min of fMRI scanning time.

### Stimuli

We used stimuli from the POPORO (pool of pairs of related objects) stimulus dataset (Kovalenko et al., 2012), which contains color images of everyday objects and animals. From these stimuli, we selected four (two animate and two inanimate) for the final study. The stimuli were selected based on (1) familiarity and visual difference, such as to maximize classification performance; and (2) accuracy and visibility scores calculated in a pilot experiment. The stimuli were presented at 50% contrast on a gray background screen. They encompassed a 4 × 4 cm square, which corresponded to a visual angle of 2.81°. The stimuli were relatively small to prevent large eye movements, which would affect our fMRI analyses. The mask was created by randomly scrambling the pixel values of all stimuli together and was also 4 × 4 cm to fully mask the presented stimuli.

### Behavioral analysis

To characterize performance on the discrimination animacy task we calculated *d*′ as the distance between the signal and the signal plus noise, calculated as the difference between the hit rate and the false alarm rate (Macmillan and Creelman, 1990). A high *d*′ value indicates better performance, and a *d*′ value of zero indicates chance-level performance.

### fMRI acquisition

Each block was scanned in a separate fMRI run, adding up to eight runs in total. In between runs, the researcher checked in with the participant and asked whether they needed a break. The experiment continued when the participant said they were ready to continue. fMRI data were recorded on a Siemens 3 T Skyra scanner with a Multiband 6 sequence (TR, 1 s; voxel size, 2 × 2 × 2 mm; TE, 34 ms) and a 32-channel head coil. For all participants, the field of view was tilted −25° from the transverse plane, using the Siemens AutoAlign Head software, resulting in the same tilt relative to the individual participant’s head position. T1-weighted structural images (MPRAGE; voxel size, 1 × 1 × 1 mm; TR, 2.3 s) were also acquired for each participant.

### fMRI preprocessing

Before decoding analyses, data were preprocessed using SPM12 (RRID:SCR_007037). All functional imaging data were motion corrected (realignment) and coregistered to the T1 structural scan. The scans were then normalized to MNI space using DARTEL (diffeomorphic anatomical registration through exponentiated lie algebra) normalization and smoothed with a 6 mm Gaussian kernel, which has been shown to improve group-level decoding accuracy (Misaki et al., 2013; Gardumi et al., 2016; Hendriks et al., 2017). A high-pass filter of 128 s was used to remove slow signal drift.

### Multivariate pattern analysis

Multivariate analyses were performed using MATLAB version 2018a (RRID:SRC_001622). We used linear discriminant analysis to decode the stimulus identity per searchlight based on the β-estimates per trial. All individual trial β-estimates were obtained from one general linear model (GLM) that contained a separate regressor for each trial set at the onset of the image [or imagery frame for imagery with a duration of 0 (spike) for the conscious and unconscious conditions and a duration of 4 for the imagery condition; Bosch et al., 2014; Dijkstra et al., 2017a,b]. Additional regressors in this GLM were (1) the animacy response screen onsets, duration set to the time until response; (2) animacy response button presses, duration 0 (spike); (3) the visibility response screen onsets, duration set to the until response; (4) visibility response button presses, duration 0 (spike); (5) onset of the first stimulus in the retro-cue task, duration 500 ms; (6) onset of the second stimulus in the retro-cue task, duration 500 ms; and (7) a constant value per run to eliminate run-specific changes in mean signal amplitude. Finally, the average signals from the white matter and CSF (Lund et al., 2005; Caballero-Gaudes and Reynolds, 2017) as well as the motion parameters were included as nuisance regressors. Decoding within and across conditions was done pairwise between all combinations of the four stimuli, resulting in six decoding pairs, over which the accuracy was then averaged. Searchlights had a radius of four voxels, resulting in 257 voxels/searchlight on average. Searchlights moved through the brain based on the center voxel such that voxels participated in multiple searchlights (Allefeld and Haynes, 2014). Leave-one-run-out cross-validation was performed, such that for each fold, a classifier was trained on three runs and tested on the fourth, left-out run. This was done for all comparisons except for imagery-conscious and imagery-unconscious cross-decoding, because these data already came from different task runs (Fig. 1). Generalization across conditions is often asymmetric, for which there could be a variety of reasons such as differences in signal-to-noise ratio between the two conditions (van den Hurck and Op de Beeck, 2019). Because we did not have a priori hypotheses about asymmetries in cross-decoding directions and because both directions revealed qualitatively similar results, we average across both cross-decoding directions before doing statistics across subjects. The names of the regions containing stimulus specific information were determined using the AAL AAL (Automated Anatomical Labeling) atlas (Tzourio-Mazoyer et al., 2002).

### Psychophysiological interaction analysis

After identifying a visual area that contained stimulus information (significant stimulus decoding) in all three conditions, we performed a psychophysiological interaction (PPI) analysis to investigate differences in connectivity between this area and the rest of the brain between the conditions (Friston et al., 1997). Per participant, the seed region was defined as an 8 mm sphere centered on the peak averaged univariate activation over the three conditions, within a 16 mm sphere centered around the voxels in which decoding was significant for all three conditions at the group level (see Fig. 3; MNI coordinates: −54, −65, −10). This approach ensures that approximately the same region was used for every participant while also taking account of differences in structural and functional anatomy between participants. This method and the size of region of interest (ROI) definition are based on recommendations in the literature for comparable analyses (Zeidman et al., 2019). One participant was excluded because the *t* contrast of the averaged activation over the three conditions versus 0 did not reach the statistical threshold of 0.05 in any of the voxels within the group sphere. The following two PPI contrasts were calculated: (conscious perception + unconscious processing) > imagery (feedforward); and (conscious perception + imagery) > unconscious processing (feedback). Connectivity with significant areas was compared in a *post hoc* analysis by calculating the difference in connectivity between each two conditions (see Fig. 4*C*; Friston et al., 1997). Note that the connectivity analyses were not stimulus specific; therefore, the first comparison, where we compare conditions that contained a mask (conscious + unconscious) with conditions that did not contain a mask (imagery), might be driven (partly) by processing of the mask instead of the stimuli preceding the mask.

### Statistical analysis

The application of standard second-level statistics, including *t* tests, to multivariate pattern analysis (MVPA) measures is in many cases invalid because of violations of assumptions. Therefore, we used permutation testing to generate the empirical null distribution, thereby circumventing the need to rely on assumptions about this distribution. We followed the approach suggested by Stelzer et al. (2013) for searchlight MVPA measurements, which uses a combination of permutation testing and bootstrapping to generate chance distributions for group studies. Because of the large computational load of searchlight decoding analysis, per participant, 25 permutation maps were generated by permuting the class labels within each run. Group-level permutation distributions were subsequently generated by bootstrapping over these 25 maps (i.e., randomly selecting one of 25 maps per participant and then averaging over participants. A total of 10,000 bootstrapping samples were used to generate the group null distribution per voxel and per comparison. The *p* values were calculated per voxel as the right-tailed area of the histogram of permutated accuracies from the mean over participants. We corrected for multiple comparisons using whole-brain false discovery rate correction with a *q* value cutoff of 0.01. Cluster correction was performed, ensuring that voxels were only identified as being significant if they belonged to a cluster of at least 50 significant voxels (Dijkstra et al., 2017a).

### Data availability

All data will be made publicly available on publication of this article. Analysis code for this study will be made available via the corresponding author on request.

## Results

### Behavioral results

**Figure 2.**
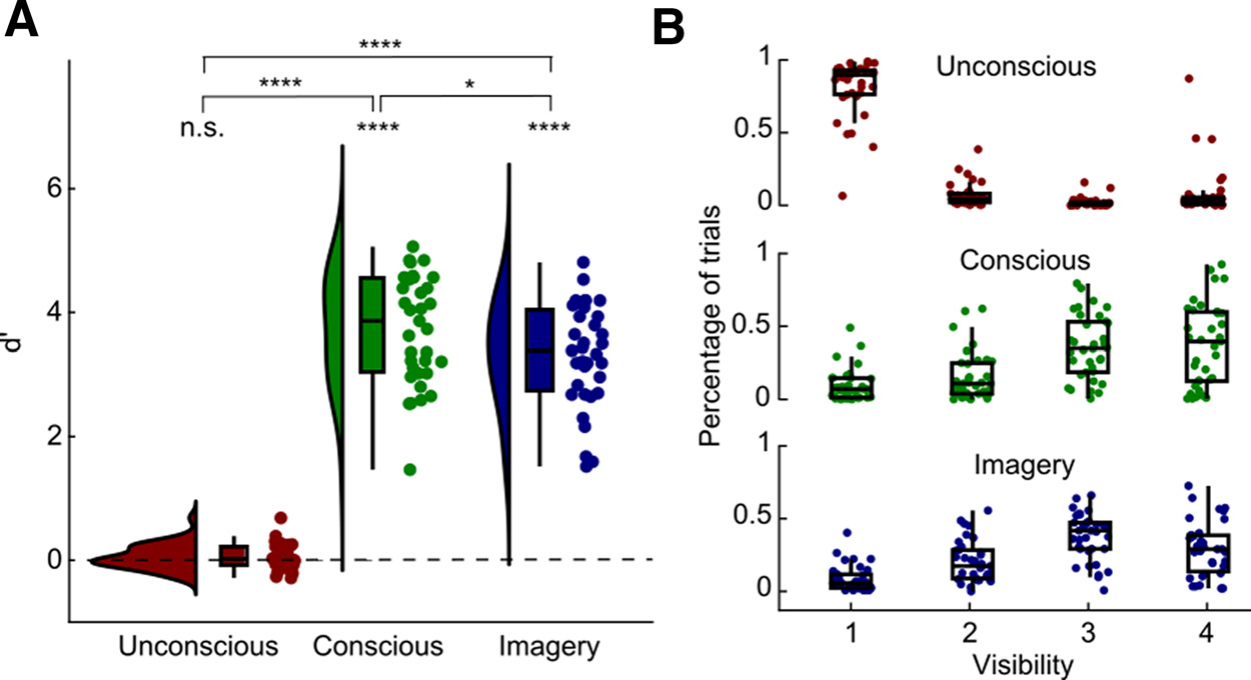
Behavioral results. ***A***, The *d*′ values for the animacy task are shown separately for each condition. The bell-shaped curves represent the distribution over participants, the boxplots indicate the four quartiles, and the dots represent individual participants. The *d*′ value was significantly higher than zero in the conscious as well as imagery condition, but not in the unconscious condition. *p < 0.05, ****p < 0.0001. ***B***, Percentage of trials of each visibility rating (1–4) separately for the three conditions. Boxplots represent the distributions over participants, and dots represent individual participants.

To check whether participants indeed did not consciously perceive the stimuli in the unconscious condition, we tested their perceptual sensitivity and visibility scores. Whereas the value of *d*′ was clearly significantly above zero for both the conscious (mean =* *3.74, SD* *=* *0.87; *t*_(34)_ = 25.40, *p *<* *0.0001) as well as the imagery (mean =* *3.32, SD* *=* *0.83; *t*_(34)_ = 23.74, *p *<* *0.0001) conditions, this was not the case for the unconscious condition (mean *= *0.05, SD* *=* *0.20; *t*_(34)_ = 1.57, *p *=* *0.127; BF01 = 0.549; Fig. 2*A*). Furthermore, the *d*′ value was significantly higher for both the conscious condition (*t*_(34)_ = 23.18, *p *<* *0.0001) and the imagery condition (*t*_(34)_ = 20.60, *p *<* *0.0001) compared with the unconscious condition. The *d*′ value in the conscious condition was also slightly higher than in the imagery condition (*t*_(34)_ = 2.62, *p *=* *0.013). Furthermore, the visibility ratings for both the conscious condition (mean =* *3.03, SD* *=* *0.54; *t*_(34)_ = 10.94, *p *<* *0.0001) as well as the imagery condition (mean =* *2.91, SD* *=* *0.38; *t*_(34)_ = 11.76, *p *<* *0.0001) were much higher than for the unconscious condition (mean =* *1.37, SD* *=* *0.54; Fig. 2*B*). A few participants rated a proportion of trials in the unconscious condition as high visibility (Fig. 2*B*); however, all of these participants still had a discrimination accuracy at chance (all <53.3%). Furthermore, there was no significant relationship between the mean visibility rating and *d*′ in the unconscious condition over participants (*r* = 0.14, *p* = 0.41). Given the nonsignificant task performance and the potential confusion caused by the randomization of response mapping between trials, these high visibility ratings during the unconscious condition are unlikely to reflect true conscious visibility. Together, these results suggest that the stimuli were indeed strongly masked, and therefore we were able to isolate feedforward processing as much as possible (Fahrenfort et al., 2007).

### Decoding within conditions

**Figure 3.**
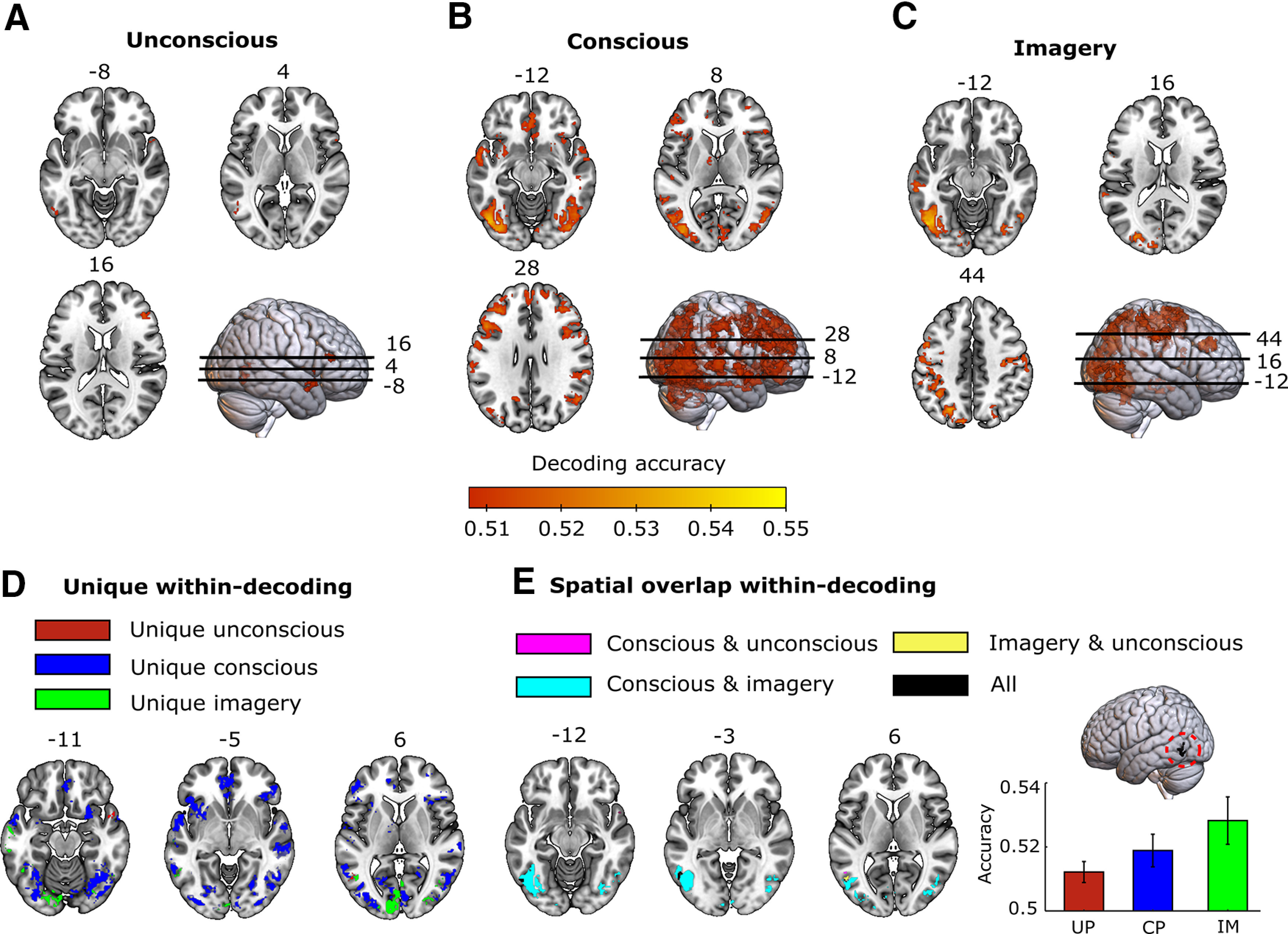
Condition-specific neural representations. ***A–C***, For each condition, significant decoding clusters are shown for various axial slices. The heatmap indicates average decoding accuracy. ***D***, ***E***, Significant decoding accuracy clusters are unique for each condition (***D***) and for spatial overlapping between conditions (***E***). Significant decoding accuracy was found in all three conditions (indicated in black, circled in red) around the left LOC at the following MNI coordinates: −54, −65, −10. Decoding accuracies for the three conditions (UP, unconscious processing; CP, conscious perception; IM, imagery) within this ROI are plotted, with the error bars indicating the standard error of the mean (SEM).

To investigate which areas represented stimulus information during the three conditions, we performed a searchlight decoding analysis separately for each condition (Fig. 3). Statistical tests were performed using group-level permutation testing as described in the study by Stelzer et al. (2013) and corrected for multiple comparisons (see Materials and Methods). Significant decoding clusters are shown in Figure 3 and are listed in Table 1. The cutoff accuracy value for significance was 0.508 for the unconscious and conscious conditions, and 0.511 for imagery. The relatively low decoding accuracy of conscious representations (∼0.55) compared with other studies (∼0.55 to 0.65; Eger et al., 2008; Axelrod and Yovel, 2015) is likely because of the backward mask, which adds noise to the stimulus response. Given the low temporal resolution of fMRI, this means that the BOLD signal at the time of the stimulus will contain a mixture of stimulus response and response to the mask, increasing variance unrelated to the stimulus and thereby decrease decoding performance. In line with previous studies (Pearson et al., 2015; Dijkstra et al., 2019), we could decode stimulus information during conscious perception as well as imagery in low- and high-level visual areas, intraparietal sulcus and lateral frontal cortex (Fig. 3*B–E*). Interestingly, significant decoding of unconscious stimuli was observed only in left high-level visual cortex, temporal pole, and lateral frontal cortex (Fig. 3*A*). There was no significant unconscious decoding in low-level visual areas. All three conditions showed stimulus representations in left LOC (Fig. 3*E*).

**Table 1:** Significant within decoding clusters

Lobe	Atlas label	Condition	MNI peak			Voxels, *N*	Peak accuracy
			*x*	*y*	*z*		
Occipital	Occipital_Sup_R	Conscious	30	−76	46	394	0.52
	Occipital_Inf_L	Conscious	−48	−70	−6	9302	0.54
		Imagery	−42	−66	−6	4922	0.54
	Occipital_Inf_R	Imagery	46	−76	−2	459	0.53
	Cuneus_L	Conscious	0	−72	34	171	0.52
	Calcarine_R	Conscious	12	−60	14	115	0.52
Temporal	Temporal_Sup_L	Conscious	−58	0	−4	951	0.53
	Temporal_Sup_R	Conscious	68	−26	2	395	0.53
			64	−2	−10	220	0.52
	Temporal_Sup_L	Imagery	−64	−38	20	100	0.53
	Temporal_Mid_L	Imagery	−60	−20	−20	182	0.53
	Temporal_Inf_L	Unconscious	−56	−62	−6	86	0.52
	Temporal_Pole_Sup_R	Unconscious	52	14	−12	91	0.52
Parietal	Parietal_Inf_L	Conscious	−32	−36	40	72	0.52
	Parietal_Inf_R	Imagery	40	−40	56	143	0.53
	Precuneus_L	Conscious	−14	−58	68	110	0.52
	Precuneus_R	Imagery	20	−72	46	284	0.53
	SupraMarginal_R	Conscious	52	−30	46	485	0.52
		Imagery	64	−22	40	90	0.52
	Cingulum_Mid_L	Imagery	−4	30	32	263	0.53
	Cingulum_Mid_R	Conscious	8	−34	42	56	0.52
Frontal	Frontal_Sup_Medial_L	Conscious	−6	58	22	468	0.52
	Frontal_Sup_R	Conscious	18	52	26	91	0.52
		Imagery	24	−4	60	172	0.53
	Frontal_Inf_Tri_L	Conscious	−48	18	28	1738	0.53
		Unconscious	44	36	16	62	0.52
	Frontal_Med_Orb_R	Conscious	2	46	−4	575	0.52
	Supp_Motor_Area_L	Imagery	−6	4	68	557	0.63
	Precentral_L	Conscious	−56	−2	26	76	0.52
		Imagery	−56	8	26	59	0.52
Cerebellum	Cerebellum_Crus2_R	Conscious	30	−80	−40	71	0.52

Atlas labels were determined using the AAL (Automated Anatomical Labeling) atlas (Tzourio-Mazoyer et al., 2002) on the basis of the MNI coordinates of the peak decoding accuracy.

### Psychophysiological interaction analysis

**Figure 4.**
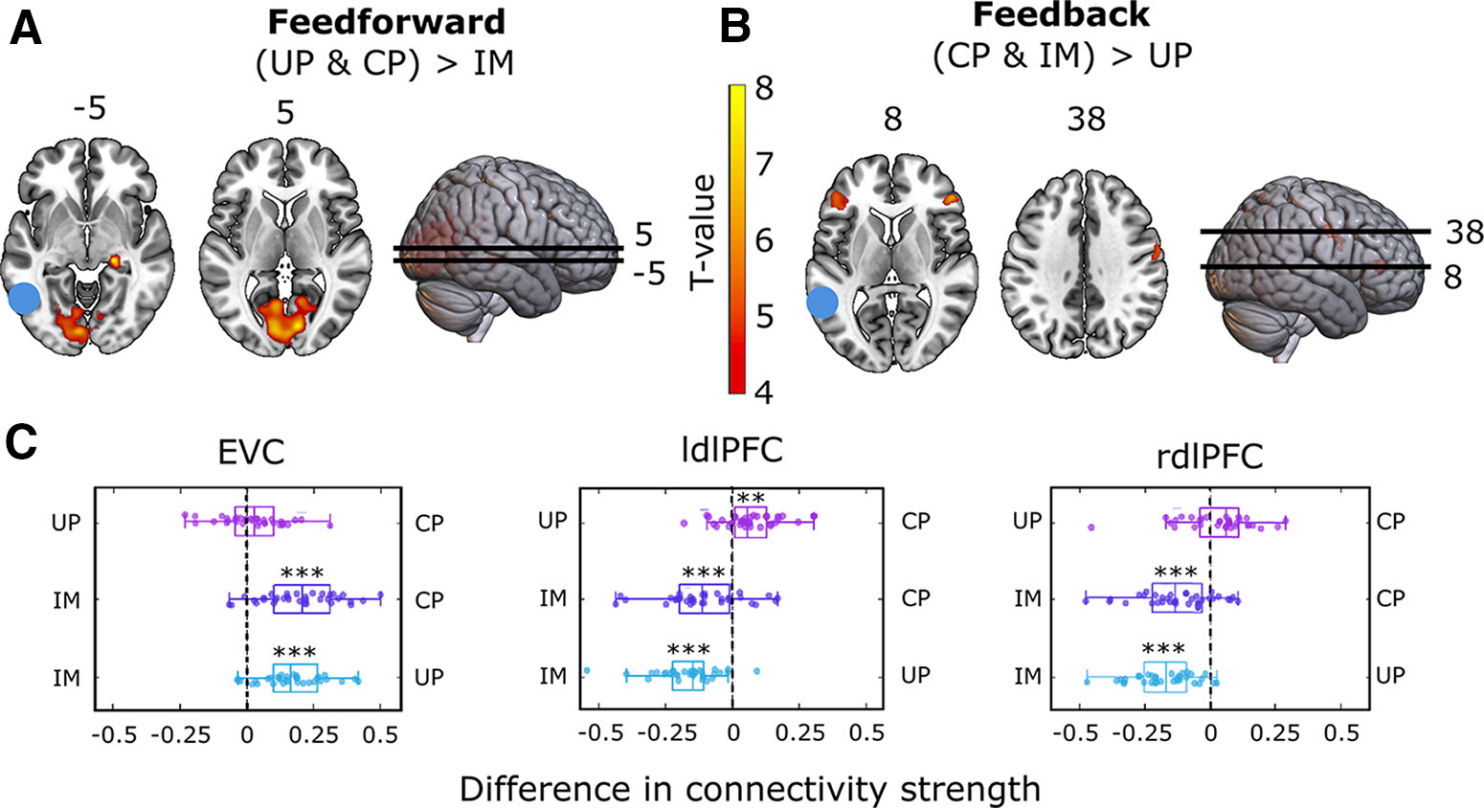
Psychophysiological interactions with left LOC as the seed region. ***A***, The blue dot illustrates the location of the seed region, red-yellow indicates brain areas that showed significantly stronger connectivity with left LOC during conscious perception (CP) and unconscious processing (UP) compared with imagery (IM; i.e., in conditions where feedforward connections were present vs in those where they were not). ***B***, The blue dot indicates the location of the seed region, red-yellow indicates brain areas that showed significantly stronger connectivity with left LOC during conscious perception and imagery compared with unconscious processing (i.e., in conditions where feedback connections were present vs in those where they were not). ***C***, Direct comparisons of connectivity between all conditions for left high-level visual cortex and EVC (left); left high-level visual cortex and left dlPFC (ldlPFC; middle); and left high-level visual cortex and right dlPFC (rdlPFC). Boxplots indicate variance over participants, and dots represent individual participants. **p < 0.005, ***p < 0.0005.

The decoding analysis showed that left LOC contained stimulus information during all three conditions (Fig. 3*E*, lateral view), suggesting that this area might be where feedback and feedforward signals overlap. Before directly investigating the representational overlap between conditions using across-condition decoding generalization, we first investigated whether this area indeed showed more feedback connectivity during conscious perception and imagery compared with unconscious processing and more feedforward connectivity during conscious and unconscious processing compared with imagery. To investigate this, we performed a PPI analysis to characterize differences in brain connectivity among the three conditions (Fig. 4, Table 2).

**Table 2:** Clusters connected with high-level within-decoding spatial overlap cluster

Lobe	Atlas label	Comparison	MNI peak			Voxels, *N*	Peak *t* value
			*x*	*y*	*z*		
Occipital	Calcarine_R	(CP and UP) > IM	10	−82	4	3654	8.21
Temporal	Temporal_Inf_L	(CP and IM) > UP	−54	−58	−8	87	5.11
Parietal	Parietal_Sup_L	(CP and IM) > UP	−22	−72	52	50	5.48
	Parietal_Sup_R	(CP and IM) > UP	16	−60	68	62	5.73
	Precuneus_L	(CP and UP) > IM	−10	−52	20	53	4.6
	Postcentral_R	(CP and IM) > UP	62	−4	36	120	5.63
Frontal	Frontal_Inf_Tri_L	(CP and IM) > UP	−46	34	8	219	5.95
	Frontal_Inf_Tri_R	(CP and IM) > UP	46	34	10	149	6.8
	Frontal_Inf_Oper_R	(CP and IM) > UP	48	4	22	60	4.66
Other	Lateral Gen Nuc	(CP and UP) > IM	22	−28	−4	80	9.12

Atlas labels determined using the AAL (Automated Anatomical Labeling) atlas (Tzourio-Mazoyer et al., 2002) on the basis of the MNI coordinates of the peak *t* value for the PPI analysis. CP, Conscious perception; UP, unconscious processing; IM, imagery.

In line with the predictions, there was stronger connectivity during conscious perception and unconscious processing compared with imagery between left LOC and EVC (MNI coordinates: −1, −85, 9) as well as right LGN (MNI coordinates: 24, −29, 4; Fig. 4*A–C*), in line with the idea that during these conditions there was more feedforward processing than during imagery. However, because these conditions also differed in whether a mask was presented (conscious and unconscious) or not (imagery), and the PPI analysis is not stimulus specific, this feedforward connectivity might partly reflect processing of the mask and not the (unconscious) stimulus before the mask. Furthermore, there was stronger connectivity during conscious perception and imagery compared with unconscious processing between left LOC and bilateral dorsolateral prefrontal cortex (dlPFC; left MNI coordinates: −45, 36, 9; right MNI coordinates: 48, 36. 9) and right lateral frontal cortex, in line with increased feedback connectivity during these conditions. *Post hoc* direct comparisons between conditions of the regions showing significant changes in connectivity (Fig. 4*A*,*B*) showed that connectivity between EVC and left LOC was stronger during conscious perception compared with imagery as well as during unconscious processing compared with imagery (Fig. 4*C*, left). Furthermore, coupling between left LOC and left dlPFC was stronger during conscious perception compared with unconscious processing as well as during imagery compared with both conscious and unconscious processing (Fig. 4*C*, middle). Finally, coupling between left LOC and right dlPFC was stronger during imagery compared with both conscious and unconscious processing (Fig. 4*C*, right). These results indicate that, in line with our assumption, long-range feedback processing is indeed stronger during conscious perception and imagery compared with unconscious processing.

### Generalization across conditions

**Figure 5.**
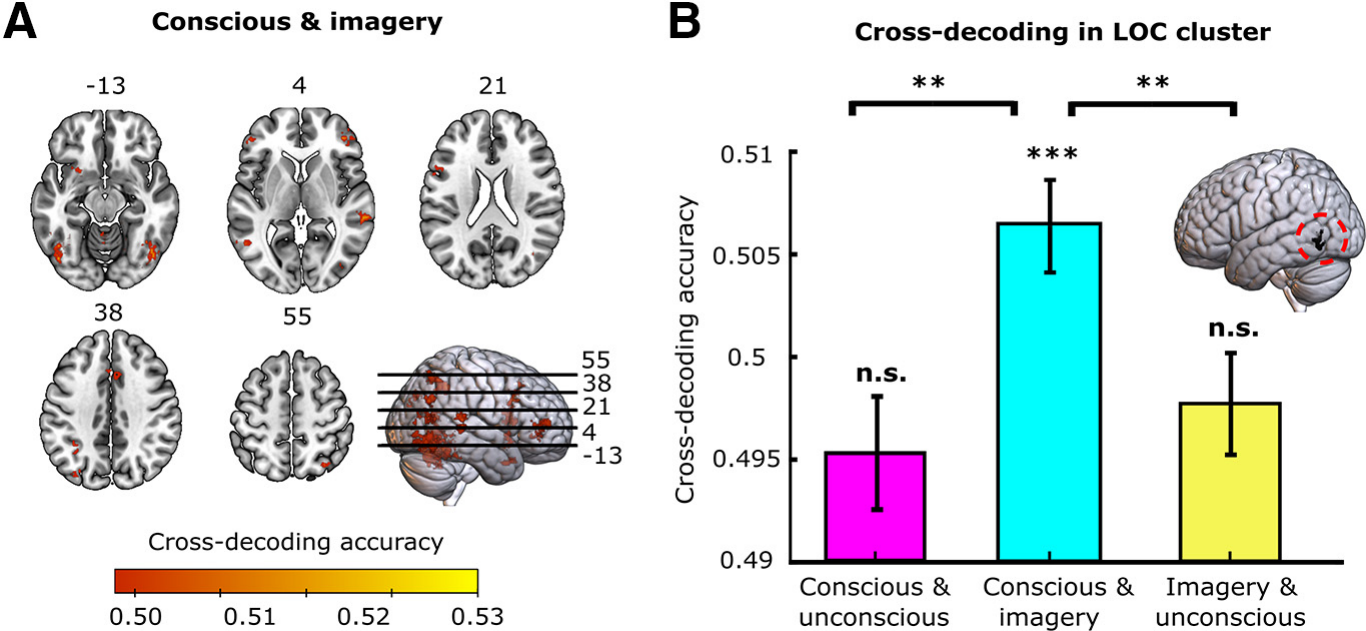
Across condition decoding accuracy. There was only significant representational overlap between conscious perception and mental imagery. ***A***, Significant cross-decoding clusters are shown for various axial slices. ***B***, Cross-decoding accuracy within the LOC cluster that had significant within-condition decoding in all three conditions (Fig. 3*E*), the same voxels were evaluated in all comparisons. Error bars indicate the SEM. n.s., Nonsignificant. *p < 0.05, **p < 0.01, ***p < 0.005.

The above decoding analysis showed that left LOC contained stimulus information during all three conditions (Fig. 3*E*, lateral view), suggesting that this area might be where feedback and feedforward signals overlap. To directly test whether the representations between conditions were similar, we performed across-condition decoding, where we trained a classifier to dissociate the stimuli in one condition and tested it in another condition. In this analysis, above-chance cross-decoding accuracy would indicate that the underlying stimulus representations are to some extent similar. Significant across-condition clusters are shown in Figure 5 and are listed in Table 3. In line with previous studies (Reddy et al., 2010; Lee et al., 2012; Pearson and Kosslyn, 2015; Dijkstra et al., 2017a, 2019), we found representational overlap between conscious perception and imagery in visual, parietal, and frontal areas (Fig. 5*A*, Table 1). In contrast, there was no significant cross-decoding between the unconscious condition and the other conditions in any brain area, suggesting an absence of representational overlap. Furthermore, despite the significant decoding in left LOC within all conditions (unconscious: mean =* *0.512, SD* *=* *0.063; conscious: mean =* *0.519, SD* *=* *0.097; imagery: mean =* *0.528, SD* *=* *0.098), there was no significant cross-decoding overlap between the unconscious condition and the other conditions in this area, even at lower statistical thresholds (Fig. 5*B*).

**Table 3:** Significant across condition decoding clusters

Lobe	Atlas label	MNI peak			Voxels, *N*	Peak accuracy
		*x*	*y*	*z*		
Occipital	Occipital_Mid_L	−38	−80	34	59	0.51
	Occipital_Inf_R	44	−78	−4	261	0.52
	Lingual_R	20	−54	−10	91	0.51
Temporal	Temporal_Mid_R	60	−34	4	122	0.52
	Temporal_Pole_Sup_L	−46	16	−26	72	0.51
	Fusiform_L	−46	−64	−18	641	0.52
Parietal	Parietal_Sup_R	32	−62	50	97	0.51
	Parietal_Inf_L	−32	−52	42	113	0.52
	Cingulum_Mid_R	4	14	30	79	0.52
	Precuneus_L	−16	−56	14	76	0.52
	Angular_R	48	−62	32	60	0.51
Frontal	Frontal_Sup_Orb_L	−26	14	−14	59	0.52
	Frontal_Mid_R	46	52	8	113	0.52
	Frontal_Inf_Oper_L	−50	12	12	183	0.52
	Frontal_Inf_Tri_L	−48	42	0	52	0.51
Cerebellum	Cerebellum_3_R	12	−38	−24	142	0.52

Atlas labels determined using the AAL (Automated Anatomical Labeling) atlas (Tzourio-Mazoyer et al., 2002) on the basis of the MNI coordinates of the peak decoding accuracy. Condition is not indicated here because only imagery-conscious across-condition decoding was significant.

Together, these results suggest that there is no representational overlap between unconscious and imagined neural representations. However, it is possible that we did not observe significant representational overlap here, not because there is no overlap, but because we do not have enough power to reveal this overlap. The results presented in Figure 5*B* show that cross-decoding accuracy between conscious perception and imagery is significantly higher than the cross-decoding accuracy between the other conditions. This means that while we cannot exclude the possibility of overlap with unconscious representations, we can conclude that representational overlap with unconscious representations is lower than the overlap between conscious and imagined representations. However, this might partly be because of the fact that unconscious representations were less strong compared with the other conditions (Fig. 3). We discuss this possibility in more detail in the Discussion.

## Discussion

In this study, we aimed to investigate the overlap between neural representations caused by feedforward versus feedback signals by comparing brain activity during mental imagery, conscious perception and unconscious processing. We found significant stimulus decoding for all three conditions in left high-level visual cortex (i.e., LOC). Furthermore, a PPI analysis showed that this area indeed showed more feedback connectivity during conscious perception and imagery compared with unconscious processing. These results suggested that this area might be the place where feedforward and feedback-initiated representations overlap. However, across-condition generalization revealed there was only significant representational overlap in this area between conscious perception and imagery, but not unconscious perception. These findings are in line with the idea that feedback changes the “format” of neural representations, leading to the reduction of overlap between representations caused by feedforward and feedback signals, but the presence of overlap between representations caused by feedback processes associated with perception of external stimuli and feedback processes associated with mental imagery.

The significant decoding of unconscious category-specific stimuli in high-level cortex agrees with previous findings (Jiang and He, 2006; Rees, 2007; Fahrenfort et al., 2012; Axelrod et al., 2015). Although both conscious and unconscious category-specific representations were present in high-level visual cortex, we did not find representational overlap between the two. This is in line with previous studies using backward masking (Bar et al., 2001) and dichoptic fusion (Schurger et al., 2010). These studies also showed conscious and unconscious representations in high-level visual cortex, but no spatial or representational overlap between them. Conscious and unconscious representations may differ in several respects, including their duration, intensity, coherence, stability, and reproducibility (Lamme and Roelfsema, 2000; Tononi and Koch, 2008; Schurger et al., 2010, 2015). It has been proposed that long-range feedback may stabilize activity in local neural processors, as if the brain “decides” what specific input it has received. The decision of the network, given the input, is what may be reflected in conscious access (Dehaene, 2014; Schurger et al., 2015). The stabilization of neural activity by feedback therefore may change the format of neural category-specific representations (Dehaene et al., 2003; King et al., 2016; Baria et al., 2017; Dijkstra et al., 2018; He, 2018; Weaver et al., 2019; Xie et al., 2020).

Although an intriguing possibility, some previous fMRI studies did report cross-decoding between conscious and unconscious conditions (Moutoussis and Zeki, 2002; Sterzer et al., 2008; Sterzer and Rees, 2008; Fahrenfort et al., 2012). In these studies, awareness of face/house stimuli was manipulated by dichoptic fusion (Moutoussis and Zeki, 2002; Fahrenfort et al., 2012), continuous flash suppression (CFS; Sterzer et al., 2008), or binocular rivalry (Sterzer and Rees, 2008). Which specific brain areas retain information about unconscious stimuli likely depends on the methods used to render the stimuli invisible (Fogelson et al., 2014; Izatt et al., 2014; Axelrod et al., 2015). Dichoptic fusion, CFS, and binocular rivalry all rely on interactions between inputs from the two eyes and may primarily affect inhibition–adaptation cycles as early as V1, although much is still unclear at present (Tong et al., 2006; Rees, 2007; Axelrod et al., 2015). In contrast, the neural effects of backward masking have previously been shown to disrupt recurrent interactions between high- and low-level visual regions (Lamme et al., 2002; Roelfsema et al., 2002; Del Cul et al., 2007; Fahrenfort et al., 2007; van Gaal and Lamme, 2012). Future research is necessary to fully determine the specific effects of each visibility manipulation on neural processing to unravel the discrepancies between studies and to understand why representational overlap between conscious and unconscious representations is sometimes observed and sometimes not.

The idea that feedback processing changes the format of neural representations suggests that the representational overlap between these different modes of perception should change over time. Because of the sluggishness of the BOLD response, fMRI lacks the temporal resolution needed to characterize such dynamics. In contrast, recent studies using methods with higher temporal resolution such as electroencephalography and magnetoencephalography (MEG) do indeed suggest differences in the timing of representational overlap among conscious perception, unconscious processing, and imagery. During conscious perception, neural representations first change rapidly over time during early time windows, likely reflecting the feedforward sweep, after which representations stabilize later in time, presumably via recurrent processing (Cichy et al., 2014; Mostert et al., 2015; Schurger et al., 2015; Baria et al., 2017; Dijkstra et al., 2018; He, 2018). Recent evidence shows that neural representations of stimuli that were strongly masked or missed during the attentional blink, only overlap with conscious conditions at early stages of input processing (until ∼250 ms; Meijs et al., 2019; Weaver et al., 2019). Furthermore, a recent MEG study revealed that representations during imagery mostly overlap with representations during later stages of conscious perception (Dijkstra et al., 2018; Xie et al., 2020). This supports the idea that neural representations of consciously reported and unreported stimuli are similar during initial feedforward (and likely local recurrent) processing, but that long-range feedback changes the neural representations, which then mimics the representations initiated by imagery-related feedback processing.

It is important to note that the exact relationship between (long-range) feedback processing and conscious awareness is still debated (Boly et al., 2017). Some theories suggest that local recurrent processing within sensory systems is sufficient for conscious experience (Lamme, 2015), whereas others propose that communication within a broader network, including frontoparietal areas, is necessary (Dehaene and Changeux, 2011; Mashour et al., 2020) and still others propose that activation of meta-representations is sufficient (Brown et al., 2019; Lau and Rosenthal, 2011). Here, we used perception rendered unconscious via backward masking as a proxy for feedforward visual processing, and in line with this assumption, our PPI results suggested that visual activity was only driven in a feedforward fashion in the unconscious condition. However, it is possible that there was still some form of feedback processing present during the unconscious condition, either weaker or more local compared with the conscious condition, that was not picked up by the PPI analysis. This means that the absence of representational overlap between the conscious and unconscious conditions might be because of other factors that are affected by awareness in addition to feedback processing. Future research directly investigating how top-down processing changes neural representations, using methods with a higher temporal resolution, will give more insight into this issue.

Finally, in line with previous studies we not only found significant cross decoding between conscious perception and imagery in several visual areas (Reddy et al., 2010; Cichy et al., 2012; Lee et al., 2012; O’Craven and Kanwisher, 2000; Albers et al., 2013; Dijkstra et al., 2017a,b), but also in parietal and frontal areas (Christophel et al., 2017; Dijkstra et al., 2017a,b). Additionally, we observed stronger connectivity between LOC and the dlPFC during imagery and conscious perception than during unconscious perception. The dlPFC has been implicated in numerous studies investigating the neural mechanisms of conscious reportability (conscious access) of input (Dehaene et al., 2006; Lau and Passingham, 2006; Rees, 2007; Davidson et al., 2010). These studies, in a similar way to ours, have all focused on conscious access of an external stimulus, whereas a recent study showed similar feedback connectivity during conscious perception and mental imagery (Dijkstra et al., 2017b). The current results suggest that dlPFC is important for conscious access, regardless of whether it is internally or externally generated. However, it should be noted that our perception task was not passive; participants actively attended to specific features of the stimulus to judge its animacy. Therefore, the overlap between imagery and perception reported here might (partly) be because of the use of similar attentional mechanisms (Dijkstra et al., 2019). During both the perception and imagery tasks, participants had to attend to specific spatial locations and features to correctly execute the animacy task. This means that during both tasks, spatial and feature-based top-down attention was used. Moreover, the increase in dlPFC connectivity during imagery compared with conscious perception might reflect the increased attentional load of generating a sensory representation in the absence of its corresponding input (Dijkstra et al., 2017a,b). Furthermore, the nature of the imagery task used here, in which the imagined image is presented relatively shortly before the imagery, might result in lingering feedforward activity. Several studies using the same paradigm only showed feedback processing during imagery (Dijkstra et al., 2017b, 2020); however, we cannot completely rule out that the imagery also contained some feedforward processing. To fully address this, future research should investigate whether similar patterns are found with conscious but passive perception and with imagery initiated from long-term memory.

An alternative possibility for our findings is that feedback does not change the representational format per se, but that during the conscious condition, feedback enhances representations of feedforward information, for example, via gain increase (Reynolds and Heeger, 2009; Wyart et al., 2012). Our results would then suggest that this kind of feedback-related enhancement is necessary to detect representational overlap between perception and imagery. This would also mean that using more sensitive methods, such as single-cell recordings, might still uncover representational overlap between the neural populations recruited during imagery and those activated by unconsciously processed stimuli.

Related to this, it is important to note that while we did find significant decoding within unconscious processing, the decoding accuracy in this condition was lower than during both imagery and conscious perception. This means that our power to detect representational overlap with the unconscious condition was lower compared with the other conditions. Therefore, we cannot rule out that our lack of representational overlap with unconscious processing is because of low unconscious decoding. It is theoretically possible that the amount of representational overlap with unconscious conditions is as high as the other conditions, but that the low power within the unconscious condition prevented us from detecting this. Low unconscious decoding may partly reflect an inherent feature of unconscious processes, in the sense that feedforward initiated representations are less strong (especially higher up in the cortical hierarchy) compared with representations that have been stabilized via long-range feedback connections as mentioned above (Lamme and Roelfsema, 2000; Tononi and Koch, 2008; Schurger et al., 2010, 2015), leading to lower decoding accuracy and therefore less power to detect representational overlap (Fahrenfort et al., 2012; van Gaal and Lamme, 2012; Weaver et al., 2019). Furthermore, although this type of masking has been shown to selectively disrupt feedback processing while keeping feedforward activity intact (Fahrenfort et al., 2007; van Gaal et al., 2008, 2011), because of the low temporal resolution of the BOLD signal we are unable to completely rule out a reduction in feedforward activity because of the masking procedure. To fully rule out this possibility, ideally, the within-decoding accuracy in all conditions is equalized experimentally, for example by lowering the contrast of the stimulus in the conscious condition (for a similar approach in behavior, see Lau and Passingham, 2006). This is an interesting avenue for future research.

In summary, our results show that neural representations measured by fMRI, triggered by purely feedforward (unconscious processing) or feedback (mental imagery) processes show reduced overlap. This suggests that the large representational overlap between imagery and perception reported in the literature (Dijkstra et al., 2019; Pearson, 2019) is undetectable for stimulus-triggered activation in the absence of feedback processing. Our results suggest that long-range feedback processing alters the format or strength of neural representations, for example, through stabilization of the neural code. More insight into this dynamic process can be gained using methods with higher temporal resolution than fMRI. Future research should explore exactly how feedback changes the format of representations and how different methods of rendering stimuli invisible affect this process.

## Synthesis

Reviewing Editor: Li Li, New York University Shanghai Decisions are customarily a result of the Reviewing Editor and the peer reviewers coming together and discussing their recommendations until a consensus is reached. When revisions are invited, a fact-based synthesis statement explaining their decision and outlining what is needed to prepare a revision will be listed below.

Both reviewers provided positive reviews and some concerns. Please address the reviewers’ concerns before the ms can be considered for publication. I summarize their comments below.

Reviewer 1

- The manuscript is well-written overall and addresses a topic of high interest. The authors have already provided some nice discussion of the limitations of their experiments. However, there are some modifications to the framing of the study, and some additional analyses that are suggested below. These points will need to be addressed before the manuscript can be published.

Main comments

- The main claim of this paper (and the title) is that representations are not overlapping between unconscious perception and imagery. This claim is not strongly supported by the data in their current form, in particular the cross-decoding analysis. As the authors note in their discussion, a key alternative reason why cross-decoding might fail is that the signal-to-noise ratio (SNR) is simply lower in the unconscious condition than conscious/imagery, as looks to be the case from Figure 3A. It is great that the discussion of this point is already included, however there are a few additional analyses that would help to clarify whether there is or is not overlap:

- Rather than just reporting the average accuracy (across all 6 of the binary classifiers), look at the pattern of decoding accuracy over individual pairs of stimuli. This could take the form of a dissimilarity matrix (a 4x4 symmetric matrix, where each element includes the decoding accuracy for one pair of stimuli being decoded).

- These matrices could be plotted side by side for the different conditions, and the structure could be compared to infer how similar the representations are. This might reveal, for instance, that there is some shared structure in the 4x4 matrix for the imagery condition and the conscious condition that is not shared with the unconscious condition. This could be explicitly tested by computing correlation coefficients between the matrices corresponding to different conditions. See for example Kriegeskorte 2008 (Frontiers in Systems Neuroscience) for examples of such an approach.

- An additional analysis, which might help to improve overall decoding accuracy and help address the first point, is to try decoding only the “animacy” of the stimulus (collapsing the rooster and fish into one group and the football and watering can into another group). Since animacy is a key feature in organizing ventral visual cortex, maybe this decoding will work better than individual stimuli. You could then do the same cross-decoding but for animacy only.

- Depending on the results of these suggested analyses, the framing of the results will need to be reconsidered. If it isn’t possible to determine whether SNR differences or true changes in representational format are responsible for the failure of cross-decoding, then other interpretations need to be considered. For instance, maybe feedback signals enhance representations of feedforward information (in the conscious perception condition) via a mechanism such as gain increase, but do not appreciably change their format. This would still lead to an interesting interpretation of the results in the context of the larger question about what factors drive the previously-reported similarity between perception and imagery. For instance, maybe the presence of some feedback-related enhancement is required in order to make it possible to detect representational “overlap” between perception and imagery. Then the conclusion of the study could be reported as something like “representational similarity between perception and imagery cannot be detected in the absence of feedback”.

- Define exactly what is meant by “overlap”. At various points in the text it seems like overlap is referring to (1) the parts of the brain where within-condition stimulus decoding is above chance for multiple conditions and (2) representations that generalize across conditions with cross-condition decoding. It would be useful to think of a second term to differentiate these (spatial overlap versus code format overlap?). Also clarify in the introduction (lines 37-47) and discussion (paragraph starting line 413) what type of overlap is being measured in those studies - is it cross-condition decoding or something else?

- In Figure 3, should include bar plots for the average values of within-condition decoding accuracy in left LOC (analogous to what is shown for cross-decoding in Figure 5B). This will make it easier to compare within-condition versus across condition decoding performance.

- Line 344 - coupling between rdlPFC and LOC was stronger for imagery than either perceptual task. This point is interesting and should be discussed further. It appears to suggest that imagery and conscious perception differ in terms of the exact nature of the feedback (both recruit feedback from ldlPFC, while only imagery recruits feedback from rdlPFC). If this is the case, how does this fit with the idea that representations are “overlapping”? Some discussion of these points is needed.

Minor points in text:

- In abstract - need to define acronym “PPI” before using it

- Line 101 - what do you mean by “counter-balanced over participants”? Does this mean the order of the runs was counter-balanced (like odd subjects do perception first, even subjects do imagery first) or does it mean something else?

- Line 121 “trial order was fully counter-balanced within participants for both tasks” - it is unclear exactly what this means.

- Are the unconscious and conscious perceptual trials interleaved in the same scan runs? If so, is the order random? I suspect so but couldn’t find this info in the methods.

- Line 219 “second comparison” - should be “first”. If you’re referring to the comparisons laid out in lines 214-216.

- Line 229 - do the 25 permutation maps each refer to one random permutated order of the class labels? If so, 25 seems like a small number of random permutations to perform (1000 is more standard). Maybe this is ok, but some justification is needed.

- In the last few sentences of abstract (lines 14-17), it sounds like the authors are referring to the conscious perception condition as purely driven by feedback (“neural representations caused by feedback...stimulus-driven (perception)”). But as noted elsewhere in the text, conscious perception includes feedback and feedforward processing. This should be clarified.

- Line 411 - “externally driven and internally driven feedback processes”. This phrase doesn’t totally make sense since feedback is always internally driven (i.e. originating from brain regions like frontal cortex). A better way to put this might be “feedback processes associated with perception of external stimuli and feedback processes associated with mental imagery”.

Reviewer 2

In the paper entitled “No overlap between unconscious and imagined representations", authors have investigated interplays of feedforward and feedback processing for neural processing during perception. Especially, authors recorded the neural responses of human participants under imagery, conscious perception and unconscious processing by using fMRI. Experimental results of this work have implied the significant similarities between imagery and conscious perception in the left lateral occipital cortex (LOC). In addition, significant connectivity between frontal area and left LOC was observed during conscious perception and imagery, not during unconscious processing.

These main results are scientifically interesting and interested in readers of eNeuro. However, in this current manuscript, the reviewer found some issues that authors should address. The reviewer requires to address these issues:

1. The reviewer recommends authors to reconsider the title of this paper. “unconscious and imagined representation” might be confused readers before reading this paper.

2. In “Participants” section, due to an accidental change, the monitor used in this experiment seemed to be different among participants (the line 90). In the current manuscript, authors claimed that this error did not to modulate the experimental results. However, the reviewer requires to indicate results of statistical tests in the performances and neural activities between these two groups.

3. Stimuli of the experiment were explained in the “Stimuli” section (the line 137). However, more details of stimuli for the experiments should be described in this section. For example, luminance values of presented stimuli, mask and background were not shown in the manuscript. Width and length of dimensions of presented stimuli and mask were not described in the current manuscript. Results of this experiments might be modulated by these experimental parameters and settings.

In addition, in various psychophysical experiments, to cancel out possible biases in the perception, mirror images were used as visual stimuli. If authors did not use mirror images in this work, please discuss possibility of biases in the perception and neural activities of overlapping.

4. In the experiments, a rooster and a football seemed to be used as the pair of visual stimuli. A fish and a watering were considered as another pair. Authors should report whether there were significant or marked differences in participants’ performance and neuronal activities between these pairs. If participants perceived them based on object categories, activated cortical regions might be distinct between these pairs.

5. Authors should clarify details of participants in more details. For example, ratio of sex for participants, states of participants’ vision (normal vision, corrected-to-normal vision), etc. It is important to describe whether participants in the experiments were naïve or trained. In addition, authors should clarify whether participants were familiar with psychophysics and fMRI researches. Furthermore, authors should clarify whether participants were aware of the aim of the experiments if they were familiar with these kinds of studies.

6. Decoding accuracy of this study for conscious representations seemed to be lower compared to previous studies (in the line 282). Authors speculated that such lower decoding accuracy was induced by the backward noise. However, the reviewer requires the additional discussion for the mechanism of interactions between decoding accuracy and backward noise. The discussions of lower decoding accuracy via backward noise potentially provide insight into the mechanism of visual perception and experimental procedures for mask noise.

7. In the line 502, the possibility of the employment of similar attentional mechanisms has been discussed the overlap between imagery and perception reported by this study. However, various kinds of visual attention for establishing and modulating visual perception have been investigated and discussed by a variety of physiological, psychophysical and computational studies (bottom-up attention, top-down attention, spatial attention, feature-based attention, object-based attention, etc). The reviewer requires that the possible attentional kinds and mechanism relating to the current study can be discussed and mentioned in the “Discussion” section.

8. In the line 102, ISI between the stimulus and the mask were set to 66 ms for the conscious condition. However, in Figure 1A, 33 ms seemed to be utilized as the SOA for CP condition. Authors must show actual ISI for their experiments in the revised manuscript.

Minor concerns:

Many abbreviations were not defined in this manuscript (ISI, GLM, CSF, ROI, MVPA, etc). Authors must define them before mention in the manuscript.

Descriptions of some terms were inconsistent during the current manuscript (d’, d prime and D’; feedforward and feed-forward).

In the line 312, “Table 3” in the main text might be “Table 2”?

In the line 461, “blinked” in the main text might be “blinded”?

## Author Response

Dear Professor Li,

We are pleased to submit a revised version of our manuscript entitled “No overlap between unconscious and imagined representations” for further consideration at eNeuro. We are grateful to both you and the reviewers for a careful reading of our paper, and the helpful set of comments. We have now reworked the manuscript and completed a revision that addresses all these points in full.

Specifically, we have:

Performed suggested alternative analyses to gain more insight into the overlap between imagery and unconscious processing;

Adjusted the title to better reflect our results from “No overlap between unconscious and imagined representations” to “No evidence for neural overlap between unconsciously processed and imagined stimuli";

Added more details about the decoding accuracy in our ROI in one of the main figures;

Added more information about experimental and statistical details;

Added a control analysis to show that changes in presentation time did not influence results;

Included more discussion on the effects of the mask on stimulus representations, the kind of attentional mechanisms that might be similar during perception and imagery and an alternative account of how feedback processing might change sensory representations.

We believe that these changes have greatly improved the manuscript.

Yours sincerely,

On behalf of the authors,

Nadine Dijkstra

Reviewer’s comments:

Reviewer 1: The manuscript is well-written overall and addresses a topic of high interest. The authors have already provided some nice discussion of the limitations of their experiments. However, there are some modifications to the framing of the study, and some additional analyses that are suggested below. These points will need to be addressed before the manuscript can be published.

Response: We thank the reviewer for their detailed and helpful comments; below we respond to each point in full.

Main comments

Comment: The main claim of this paper (and the title) is that representations are not overlapping between unconscious perception and imagery. This claim is not strongly supported by the data in their current form, in particular the cross-decoding analysis. As the authors note in their discussion, a key alternative reason why cross-decoding might fail is that the signal-to-noise ratio (SNR) is simply lower in the unconscious condition than conscious/imagery, as looks to be the case from Figure 3A. It is great that the discussion of this point is already included, however there are a few additional analyses that would help to clarify whether there is or is not overlap:

Response: We agree with the reviewer that we cannot be sure that there is no overlap between unconscious and imagined stimuli. We appreciate the suggestions for analyses to further investigate this claim, but, as discussed in more detail below, none of these were able to provide new evidence. In light of this, we have changed the title of the paper to: “No evidence for neural overlap between unconsciously processed and imagined stimuli”. We feel like this more accurately describes the results that we want to communicate to the field.

Comment: Rather than just reporting the average accuracy (across all 6 of the binary classifiers), look at the pattern of decoding accuracy over individual pairs of stimuli. This could take the form of a dissimilarity matrix (a 4x4 symmetric matrix, where each element includes the decoding accuracy for one pair of stimuli being decoded).

These matrices could be plotted side by side for the different conditions, and the structure could be compared to infer how similar the representations are. This might reveal, for instance, that there is some shared structure in the 4x4 matrix for the imagery condition and the conscious condition that is not shared with the unconscious condition. This could be explicitly tested by computing correlation coefficients between the matrices corresponding to different conditions. See for example Kriegeskorte 2008 (Frontiers in Systems Neuroscience) for examples of such an approach.

Response: We thank the reviewer for this helpful suggestion and have performed the suggested analysis. We selected the voxels in LOC that showed significant decoding within each condition (black region in Fig. 3E) ¬¬and plotted the pairwise decoding accuracies for each condition, averaged over participants.

However, note that there are only 6 pairwise comparisons, which is not enough to calculate reliable correlations. Therefore, while an interesting and potentially insightful analysis approach, our data structure is not optimized for this analysis and it can therefore not provide new insights here.

Comment: An additional analysis, which might help to improve overall decoding accuracy and help address the first point, is to try decoding only the “animacy” of the stimulus (collapsing the rooster and fish into one group and the football and watering can into another group). Since animacy is a key feature in organizing ventral visual cortex, maybe this decoding will work better than individual stimuli. You could then do the same cross-decoding but for animacy only.

Response: This is an interesting suggestion, but unfortunately, our design was also not optimized for this analysis. For the retro-cue paradigm with which imagery was implemented to work in an unbiased way, it is paramount that the order of the preceding stimuli and the retro-cue are balanced perfectly. We did not do this for animacy, which means that any overlap with imagery when purely decoding accuracy, could simply be the result of spill-over BOLD from the cue images preceding the retro-cue.

Comment: Depending on the results of these suggested analyses, the framing of the results will need to be reconsidered. If it isn’t possible to determine whether SNR differences or true changes in representational format are responsible for the failure of cross-decoding, then other interpretations need to be considered. For instance, maybe feedback signals enhance representations of feedforward information (in the conscious perception condition) via a mechanism such as gain increase, but do not appreciably change their format. This would still lead to an interesting interpretation of the results in the context of the larger question about what factors drive the previously-reported similarity between perception and imagery. For instance, maybe the presence of some feedback-related enhancement is required in order to make it possible to detect representational “overlap” between perception and imagery. Then the conclusion of the study could be reported as something like “representational similarity between perception and imagery cannot be detected in the absence of feedback”.

Response: We thank the reviewer for suggesting this alternative explanation and have added the following paragraph in the discussion. On Page 20, line 8:

"To fully address this, future research should investigate whether similar patterns are found with conscious but passive perception and with imagery initiated from long-term memory.

Furthermore, an alternative possibility for our findings is that feedback does not change the representational format per se, but that during the conscious condition, feedback enhances representations of feedforward information, for example via gain increase (Reynolds & Heeger, 2009; Wyart, Nobre, & Summerfield, 2012). Our results would then suggest that this kind of feedback-related enhancement is necessary to detect overlap between perception and imagery. This would also mean that using more sensitive methods, such as single-cell recordings, might still uncover overlap between the neural populations recruited during imagery and those activated by unconsciously processed stimuli.

Related to this, it is important to note that while we did find significant decoding within unconscious processing, the decoding accuracy in this condition was lower than during both imagery and conscious perception.”

And adjusted the following in the concluding paragraph on page 21, line 5:

"This suggests that the large representational overlap between imagery and perception reported in the literature (Dijkstra, Bosch, & van Gerven, 2019; Pearson, 2019) is undetectable for stimulus triggered activation in the absence of feedback processing. Our results suggest that long-range feedback processing alters the format or strength of neural representations, for example through stabilization of the neural code.”

Comment: Define exactly what is meant by “overlap”. At various points in the text it seems like overlap is referring to (1) the parts of the brain where within-condition stimulus decoding is above chance for multiple conditions and (2) representations that generalize across conditions with cross-condition decoding. It would be useful to think of a second term to differentiate these (spatial overlap versus code format overlap?). Also clarify in the introduction (lines 37-47) and discussion (paragraph starting line 413) what type of overlap is being measured in those studies - is it cross-condition decoding or something else?

Response: We agree that this ambiguity with respect to the kind of overlap is confusing. We have adopted the suggested term of ‘spatial overlap’ for information in the same location in the brain and ’representational overlap’ for significant cross-decoding and have adjusted this in the text.

Comment: In Figure 3, should include bar plots for the average values of within-condition decoding accuracy in left LOC (analogous to what is shown for cross-decoding in Figure 5B). This will make it easier to compare within-condition versus across condition decoding performance.

Response: We have added these bars in the figure.

Comment: Line 344 - coupling between rdlPFC and LOC was stronger for imagery than either perceptual task. This point is interesting and should be discussed further. It appears to suggest that imagery and conscious perception differ in terms of the exact nature of the feedback (both recruit feedback from ldlPFC, while only imagery recruits feedback from rdlPFC). If this is the case, how does this fit with the idea that representations are “overlapping”? Some discussion of these points is needed.

Response: We thank the reviewer for pointing this out. We are a bit hesitant to interpret the specific pattern of right versus left dlPFC recruitment during imagery versus perception because this analysis only allows us to quantify differences between conditions, and not recruitment per se: so the fact that the connection between LOC and right dlPFC was not significantly stronger during the conscious condition compared to the unconscious condition, does not mean it was not present. However, we can still conclude that imagery led to stronger connectivity with dlPFC than conscious perception in both hemispheres. To highlight this difference, we have added the following to the discussion on page 20:

"The current results suggest that dlPFC is important for conscious access, regardless of whether it is internally or externally generated. However, it should be noted that our perception task was not passive; participants actively attended to specific features of the stimulus in order to judge its animacy. Therefore, the overlap between imagery and perception reported here might (partly) be due to the employment of similar attentional mechanisms (Dijkstra et al., 2019). Moreover, the increase in dlPFC connectivity during imagery compared to conscious perception might reflect the increased attentional load of generating a sensory representation in the absence of its corresponding input (Dijkstra et al, 2017).”

Minor points in text

Comment: In abstract - need to define acronym “PPI” before using it.

Response: we have changed this in the text.

Comment: Line 101 - what do you mean by “counter-balanced over participants”? Does this mean the order of the runs was counter-balanced (like odd subjects do perception first, even subjects do imagery first) or does it mean something else?

Response: Exactly, the order of the runs was counterbalanced. We have clarified this in the text:

Line 101: “The experiment consisted of two tasks, a perception and an imagery task, which were executed in interleaved blocks and whether participants started with the imagery or perception task was counterbalanced over participants.”

Comment: Line 121 “trial order was fully counter-balanced within participants for both tasks” - it is unclear exactly what this means.

Response: We clarified this as follows: “The order of the different stimuli and SOAs (unconscious versus conscious trials) within the perception task and the stimuli and retro-cue combinations during imagery was fully counterbalanced within participants.”

Comment: Are the unconscious and conscious perceptual trials interleaved in the same scan runs? If so, is the order random? I suspect so but couldn’t find this info in the methods.

Response: We have clarified this in the comment above.

Comment: Line 219 “second comparison” - should be “first”. If you’re referring to the comparisons laid out in lines 214-216.

Response: Thank you for noticing, we have adjusted this in the text.

Comment: Line 229 - do the 25 permutation maps each refer to one random permutated order of the class labels? If so, 25 seems like a small number of random permutations to perform (1000 is more standard). Maybe this is ok, but some justification is needed.

Response: We did indeed only perform 25 permutation maps per participant, in line with the recommendations from Stelzer et al. 2013. Note that each permutation for each participant reflects a whole-brain searchlight decoding analysis, which is very computationally demanding. To accommodate this, Stelzer et al. came up with this method in which a low number of within-subject permutations are made but the group null-distribution is then based on 10000 bootstrapping samples in which a random map is drawn per participant and then averaged over participants. We have changed this in the text to clarify this as follows:

"We followed the approach suggested by (Stelzer, Chen, & Turner, 2013) for searchlight MVPA measurements which uses a combination of permutation testing and bootstrapping to generate chance distributions for group studies. Due to the large computational load of searchlight decoding analysis, per participant, 25 permutation maps were generated by permuting the class labels within each run. Group-level permutation distributions were subsequently generated by bootstrapping over these 25 maps: i.e. randomly selecting one out of 25 maps per participant and then averaging over participants. 10000 bootstrapping samples were used to generate the group null-distribution per voxel and per comparison. P-values were calculated per voxel as the right-tailed area of the histogram of permutated accuracies from the mean over participants.”

Comment: In the last few sentences of abstract (lines 14-17), it sounds like the authors are referring to the conscious perception condition as purely driven by feedback (“neural representations caused by feedback...stimulus-driven (perception)”). But as noted elsewhere in the text, conscious perception includes feedback and feedforward processing. This should be clarified.

Response: We have changed this as follows:

"Neural representations influenced by feedback, either stimulus-driven (perception) or purely internally-driven (imagery), are however relatively similar.”

Comment: Line 411 - “externally driven and internally driven feedback processes”. This phrase doesn’t totally make sense since feedback is always internally driven (i.e. originating from brain regions like frontal cortex). A better way to put this might be “feedback processes associated with perception of external stimuli and feedback processes associated with mental imagery”.

Response: we have changed this as suggested.

MCD Medical

Reviewer 2

Comment: In the paper entitled “No overlap between unconscious and imagined representations", authors have investigated interplays of feedforward and feedback processing for neural processing during perception. Especially, authors recorded the neural responses of human participants under imagery, conscious perception and unconscious processing by using fMRI. Experimental results of this work have implied the significant similarities between imagery and conscious perception in the left lateral occipital cortex (LOC). In addition, significant connectivity between frontal area and left LOC was observed during conscious perception and imagery, not during unconscious processing.

These main results are scientifically interesting and interested in readers of eNeuro. However, in this current manuscript, the reviewer found some issues that authors should address. The reviewer requires to address these issues:

Response: We thank the reviewer for their helpful comments and respond to each point in full below.

Comment 1. The reviewer recommends authors to reconsider the title of this paper. “unconscious and imagined representation” might be confused readers before reading this paper.

Response: We agree with the reviewer that the title in its current form is unclear and have changed it to: “No evidence for neural overlap between unconsciously processed and imagined stimuli”.

Comment 2. In “Participants” section, due to an accidental change, the monitor used in this experiment seemed to be different among participants (the line 90). In the current manuscript, authors claimed that this error did not to modulate the experimental results. However, the reviewer requires to indicate results of statistical tests in the performances and neural activities between these two groups.

Response: We have added statistical comparisons between these two groups for behavioural performance:

"Due to an accidental change in the refresh rate of the monitor (from 60 Hz to 75 Hz) the timing was slightly different for 6/35 participants: presentation from 17ms to 13ms, ISI conscious from 66ms to 80ms, so that the presentation times were slightly shorter for the unconscious condition and slightly longer for the conscious condition. Because this error did not change visibility ratings (unconscious: 1.37 (SD = 0.27) versus 1.35 (SD = 0.58); t(33) = 0.079, p = 0.94 - conscious: 2.92 (SD = 0.37) vs 2.98 (SD = 0.61); t(33) = -0.25, p = 0.80) or discrimination sensitivity (unconscious: 0.19 (SD = 0.28) versus 0.03 (SD = 0.18); t(33) = 1.9, p = 0.07 - conscious: 3.33 (SD = 0.61) vs 3.82 (SD = 0.90); t(33) = -1.26, p = 0.22) we decided not to remove these participants.”

Note that, if anything, the difference for the unconscious d’ is in the opposite direction as to what would be expected if the change in presentation time did really alter perception: the d’ was numerically higher (0.19) for the 6 participants with shorter presentation times (13ms) than for the others (0.03, 17ms). Furthermore, it is important to note that, due to the low number of participants (6/35), these statistical tests are underpowered, especially for such noisy estimates as neural decoding analyses. Moreover, for our research question, the most important thing was that these differences in SOA did not lead to differences in conscious perception (e.g. made the unconscious condition suddenly conscious or vice versa), which the behavioural analyses show was not the case. Together, this made us decide against running additional neural comparisons.

Comment 3. Stimuli of the experiment were explained in the “Stimuli” section (the line 137). However, more details of stimuli for the experiments should be described in this section. For example, luminance values of presented stimuli, mask and background were not shown in the manuscript. Width and length of dimensions of presented stimuli and mask were not described in the current manuscript. Results of this experiments might be modulated by these experimental parameters and settings.

In addition, in various psychophysical experiments, to cancel out possible biases in the perception, mirror images were used as visual stimuli. If authors did not use mirror images in this work, please discuss possibility of biases in the perception and neural activities of overlapping.

Response: We agree with the reviewer that more details on the stimuli should be reported and have added the following:

"We used stimuli from the POPORO stimulus data set (Kovalenko, Chaumon, & Busch, 2012), which contains colour images of everyday objects and animals. From these stimuli we selected four (two animate and two inanimate) for the final study. The stimuli were selected based on (a) familiarity and visual difference, such as to maximise classification performance and on (b) accuracy and visibility scores calculated in a pilot experiment. The stimuli were presented at 50% contrast on a grey background screen. They encompassed a 4 by 4 cm square which corresponded to a visual angle of 2.81 degrees. The stimuli were relatively small to prevent large eye-movements, which would affect our fMRI analyses. The mask was created by randomly scrambling the pixel values of all stimuli taken together and was also 4 by 4 cm in order to fully mask the presented stimuli.”

Unfortunately, we did not measure exact luminance values in this experiment and are therefore unable to report these details.

Comment 4. In the experiments, a rooster and a football seemed to be used as the pair of visual stimuli. A fish and a watering were considered as another pair. Authors should report whether there were significant or marked differences in participants’ performance and neuronal activities between these pairs. If participants perceived them based on object categories, activated cortical regions might be distinct between these pairs.

Response: The stimuli were not divided based on separate pairs, but all stimuli were compared against each other. To clarify this in the manuscript, we have added the following line to the legend of Figure 1:

"(C) Stimuli used: a rooster, a football, a fish and a watering can from the POPORO stimulus data set (Kovalenko, Chaumon, & Busch, 2012). The neural analyses focused on pairwise comparisons between all possible combinations of stimuli.”

Comment 5. Authors should clarify details of participants in more details. For example, ratio of sex for participants, states of participants’ vision (normal vision, corrected-to-normal vision), etc. It is important to describe whether participants in the experiments were naïve or trained. In addition, authors should clarify whether participants were familiar with psychophysics and fMRI researches. Furthermore, authors should clarify whether participants were aware of the aim of the experiments if they were familiar with these kinds of studies.

Response: We thank the reviewer for pointing out this omission and have added some information to the participants section as follows:

"Thirty-seven participants with normal or corrected-to-normal vision gave written informed consent and participated in the study. All participants were naïve to the aim of the experiment and most participants were familiar with similar visual perception fMRI studies. “

As per the GDPR agreement, because we did not have any apriori hypothesis about sex differences, we did not enquire participant’s sex and are therefore unable to report the sex ratio of this study.

Comment 6. Decoding accuracy of this study for conscious representations seemed to be lower compared to previous studies (in the line 282). Authors speculated that such lower decoding accuracy was induced by the backward noise. However, the reviewer requires the additional discussion for the mechanism of interactions between decoding accuracy and backward noise. The discussions of lower decoding accuracy via backward noise potentially provide insight into the mechanism of visual perception and experimental procedures for mask noise.

Response: We agree with the reviewer that this warrants more discussion have added the following to describe this in more depth page 10, line 11:

"The relatively low decoding accuracy of conscious representations (∼0.55) compared to other studies (∼0.55-0.65) (e.g. Eger et al., 2008; Axelrod & Yovel, 2015) is likely due to the backward mask, which adds noise to the stimulus response. Given the low temporal resolution of fMRI, this means that the BOLD signal at the time of the stimulus will contain a mixture of stimulus response and response to the mask, increasing variance unrelated to the stimulus and thereby decrease decoding performance.”

Comment 7. In the line 502, the possibility of the employment of similar attentional mechanisms has been discussed the overlap between imagery and perception reported by this study. However, various kinds of visual attention for establishing and modulating visual perception have been investigated and discussed by a variety of physiological, psychophysical and computational studies (bottom-up attention, top-down attention, spatial attention, feature-based attention, object-based attention, etc). The reviewer requires that the possible attentional kinds and mechanism relating to the current study can be discussed and mentioned in the “Discussion” section.

Response: We agree that this is ambiguous and have added the following to that specific section in the discussion to clarify:

"Therefore, overlap between imagery and perception reported here might (partly) be due to the employment of similar attentional mechanisms (Dijkstra et al., 2019). During both the perception and imagery task, participants had to attend to specific spatial locations and features in order to correctly execute the animacy task. This means that during both tasks, spatial and feature based top-down attention was employed.”

Comment 8. In the line 102, ISI between the stimulus and the mask were set to 66 ms for the conscious condition. However, in Figure 1A, 33 ms seemed to be utilized as the SOA for CP condition. Authors must show actual ISI for their experiments in the revised manuscript.

Response: We thank the reviewer for noticing this discrepancy and have adjusted it in the figure.

Minor concerns:

Comment: Many abbreviations were not defined in this manuscript (ISI, GLM, CSF, ROI, MVPA, etc). Authors must define them before mention in the manuscript.

Response: We thank the reviewer for pointing this out and have adjusted this in the text so that the full term is spelled out at the first mention of each abbreviation.

Comment: Descriptions of some terms were inconsistent during the current manuscript (d’, d prime and D’; feedforward and feed-forward).

Response: We have adjusted this in the manuscript (always d’, and always feedforward).

Comment: In the line 312, “Table 3” in the main text might be “Table 2”?

Response: Indeed, we changed this.

Comment: In the line 461, “blinked” in the main text might be “blinded”?

Response: Some consciousness researchers refer to stimuli that are not perceived during the attentional blink, as ‘blinked’. However, we have rephrased this in the manuscript because it was indeed ambiguous.

"Recent evidence shows that neural representations of stimuli that were strongly masked or missed during the attentional blink, only overlap with conscious conditions at early stages of input processing (until ∼250ms; Meijs, Mostert, Slagter, de Lange, & van Gaal, 2019; Weaver et al., 2019).”
